# Enhancing knowledge mastery in resident students through peer-teaching: a study in respiratory medicine

**DOI:** 10.1186/s12909-024-05130-w

**Published:** 2024-03-29

**Authors:** Chen Zhu, Heshen Tian, Fugui Yan, Jing Xue, Wen Li

**Affiliations:** 1 Department of Respiratory and Critical Care Medicine Key Laboratory of Respiratory Disease of Zhejiang Province, The Second Affiliated Hospital of Zhejiang University School of Medicine, Hangzhou, 310009 Zhejiang China; 2 Department Rheumatology, The Second Affiliated Hospital of Zhejiang University School of Medicine, Hangzhou, 310009 Zhejiang China; 3 Department Education, The Second Affiliated Hospital of Zhejiang University School of Medicine, Hangzhou, 310009 Zhejiang China

**Keywords:** Resident students, Peer-assisted learning, Near-peer assisted learning, Knowledge mastery

## Abstract

**Aim:**

The transition from medical students to competent physicians requires comprehensive training during residency programs. In China, resident students typically undergo 2- or 3-year training programs. While they learn from patient interactions under the guidance of experienced doctors, integrating theoretical knowledge from textbooks into practical cases remains a challenge. This study aimed to explore the impact of medical interns acting as peer-students on the knowledge mastery of resident students.

**Method:**

The participants of this study consisted of resident students specializing in respiratory medicine at the Second Affiliated Hospital of Zhejiang University, School of Medicine. Resident students were given the opportunity to volunteer as peer-teachers for medical interns in the respiratory department. Those who chose to instruct interns were automatically placed into the test group, while those who opted not to partake in intern instruction formed the control group. In their role as peer-teachers, resident students assumed the responsibility of guiding interns in patient management throughout the entire continuum, spanning from initial engagement to discharge, a commitment that extended over a minimum period of 2 weeks. The resident students' academic performance was evaluated through a departmental examination consisting of 50 multiple-choice questions, which was administered upon completing their rotation. Statistical analysis was performed to assess the impact of peer-teaching on the resident students’ performance.

**Results:**

Between January 2023 and June 2023, a total of 158 resident students completed their rotation in the respiratory department. Among them, 40 resident students willingly took on the responsibility of instructing medical interns, while 118 resident students did not participate in intern teaching. With a "one-to-one" teaching policy in place, the overall satisfaction rate of the interns was an impressive 95.35%. Pre-rotation test scores for the test group averaged 81.66 ± 8.325 (Mean ± SD) and the control group averaged 81.66 ± 8.002, without significance. The departmental examination scores of the test group averaged 85.60 ± 7.886, while the control group scored an average of 82.25 ± 8.292, with a statistically significant difference (*p* = 0.027).

**Conclusion:**

In conclusion, our study underscores the positive influence of peer-teaching on the knowledge mastery of resident students.

## Introduction

The multifaceted role of physicians has evolved beyond that of mere illness curers; it now encompasses that of a skilled practitioner, a medical scholar, and a medical educator. Junior physicians embark on a rigorous five-year undergraduate education, laying the foundation for their medical careers. However, the chasm between theory and real-world practice becomes evident when they begin engaging with patients. Thus, clinical medical education assumes paramount importance in the growth and development of physicians. In China, a transformative approach known as the residency training program [1]. has been implemented to bridge this gap.

Introduced in 2014, the residency training program aims to enhance the medical knowledge and clinical skills of junior physicians. Over the course of 2 to 3 years, trainees undergo rotations in various departments of internal medicine, including emergency, intensive care, and anesthesia. Unlike their time in medical school, resident students now take charge of managing patients from admission to discharge under the supervision of experienced physicians, transcending the realm of textbook learning. However, due to limited exposure to diverse cases during their rotations, cultivating comprehensive competency in junior physicians remains a challenge [2, 3]. To address this, senior physicians actively impart their knowledge through various methods, including regular lectures, bedside teaching, ward observation, and case study discussions. Nevertheless, these traditional approaches predominantly adopt a teacher-guided format, leading to passive knowledge reception among residents with uncertain efficacy [2, 4]. Consequently, some residents may struggle to demonstrate strong knowledge mastery, leading to difficulties in passing the final examination.

As the landscape of medical education continually evolves, it is imperative to identify innovative and effective teaching methodologies to elevate the capabilities of resident physicians. In the pursuit of more effective teaching methods, medical education has witnessed the emergence of innovative approaches, such as problem-based learning (PBL) [5]. team-based learning (TBL) [6]. and case-based learning (CBL) [7]. These methodologies have successfully shifted the dynamics from traditional lecture-based formats to student-guided, interactive platforms, fostering a sense of enthusiasm among resident students [8]. Unsurprisingly, students have shown a preference for these dynamic learning methods, which have demonstrated promising results in enhancing knowledge acquisition.

However, despite their advantages, these methods are not without their limitations. The reliance on high-quality scripted materials and the time-consuming nature of implementation have hindered their continuous adoption. In light of these challenges, educators have turned their attention to peer-assisted learning (PAL) as a potential solution [9]. PAL empowers medical students to take charge of their own learning with the support and stimulation of their peers. PAL has gained recognition for its effectiveness in medical education, with numerous studies demonstrating its benefits in elevating scores and clinical skills [10]. as well as stimulating enthusiasm for learning. Huang et al. [11]. have highlighted the positive impact of PAL, showing improved performance in Objective Structured Clinical Skills Examination (OSCE) tests and overall academic and clinical skills among participants. Notably, PAL has proven to be particularly suitable for clinical students compared to their preclinical counterparts [12].

Recognizing the potential of PAL to mobilize enthusiasm and enhance learning outcomes, we sought to apply this approach in the training of resident students. Additionally, we identified undergraduates, particularly interns, as key participants in patient management, often in need of guidance while navigating their clinical experiences. In response, we devised a unique "one-to-one" policy rooted in PAL principles. This policy encourages collaborative learning through the formation of small medical groups comprising one undergraduate and one resident student. Undergraduates gain valuable patient management experiences while benefiting from consultations with relatively "senior" physicians. On the other hand, resident students may be motivated to elevate their learning as they engage with and support undergraduates [13]. To assess the effectiveness of this novel policy, we have incorporated a bidirectional evaluation system, whereby both resident students and undergraduates provide feedback upon completing their rotations. This evaluation will serve as a crucial measure in validating the efficiency and impact of the "one-to-one" policy in our department.

Through the implementation and continuous refinement of this policy, our aim is to explore its influence on knowledge mastery in resident students. By embracing the potential of PAL and fostering a collaborative learning environment, we strive to cultivate competent, enthusiastic, and empathetic physicians who are well-prepared to meet the challenges of modern healthcare. As we delve into the outcomes and experiences of this initiative, we seek to contribute valuable insights to the broader landscape of medical education, further refining the training of future generations of medical professionals.

## Methods

### Participants

This study involved resident students and undergraduates participating in the respiratory medicine rotation at the Second Affiliated Hospital of Zhejiang University School of Medicine between January 2023 and June 2023.

For peer-teachers, participants should be resident students who majored in internal medicine, emergency, critical care or anesthesia. For peer-students, participants should be interns enrolled in either the five-year program (normal program) or the eight-year program (advanced program with more basic medical education in the first two years) at Zhejiang University School of Medicine. The cohort included a total of 158 resident students and 43 interns, all meeting the criteria.

The recruitment process took place during the admission education sessions for both resident students and interns at the first day in our department. Resident students and interns collaboratively exercised their preferences to establish small medical groups on a voluntary basis. The formation of these small medical groups occurred through dual choices and was formalized through the signing of an agreement.

**Fig. 1:**
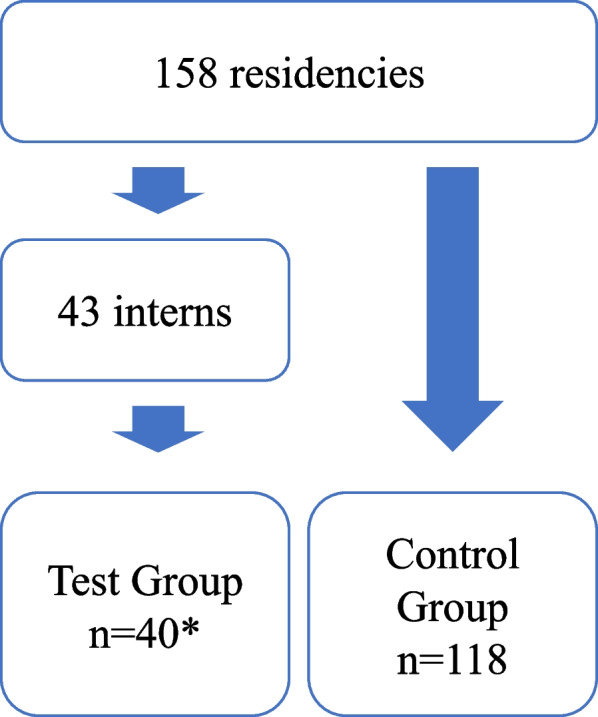
Design and process of the study. * 3 resident students instructed 2 interns at different time during their rotation

All resident students who coupled interns as peer-teachers were enrolled as test group, and the rest of resident students (free from interns’ instruction) were enrolled as control group. After the recruitment, 40 resident students participated in the policy to instruct 43 interns. Notably, three resident students took on the role of instructors for two interns each, although their instructional sessions occurred at different times. The enrollment process is represented in Fig. 1, and baseline statistics are shown in Table 1.

**Table 1:** The Baseline Characteristics of resident students

Characteristics	All (*n* = 158)	Test Group (*n* = 40)	Control Group (*n* = 118)	*P* value*
Sex, male, Frequency (Percentage)	58 (36.7)	13 (32.5)	45 (38.1)	0.52
Age, yr	26.76 ± 2.93	26.98 ± 3.33	26.68 ± 2.78	0.58
Grade in residency training program				
1st year	108	25	83	0.35
2nd year and above	50	15	35	
Specialty				
Internal Medicine	68	20	48	0.30
Not Internal Medicine	90	20	70	
Education background				
Doctorate	38	11	27	0.52
Master degree	9	1	8	
Bachelor degree**	111	28	83	
Whether Master candidate				
Yes	36	8	28	0.63
No	122	32	90	

^*^ *P* value is represented as Test group vs. Control group

^**^Master candidate is included

### Procedures

Upon establishment, each resident student-intern medical couple was officially registered, and an agreement with signs of each side was filled out. The resident students in the coupled group assumed the role of instructors for their paired interns during their daily interactions with patients and families. This comprehensive engagement encompassed academic explanations (such as questioning and co-learning in textbooks or guidelines, etc.), guidance on medical procedures (such as prescription, skills and practice, and medical documentation, etc.), analysis of ancillary examinations (such as blood tests, electrocardiogram, ultrasonography and radiological examinations), and initial clinical decision-making. A valid instruction should meet following criteria: 1) Duration: The instruction should span a minimum of 10 weekdays. 2) Daily Duration: The instructor should dedicate no less than 2 h per day to the instructional sessions. 3) Patient Engagement: The instruction should involve at least 5 patients during the specified period. All the peer-teachers participated in our policy met these criteria in our study.

Questionnaires were administered independently to each coupled resident student-intern pair before departure of either participant. The questionnaire design drew inspiration from published research [14]. incorporating minor modifications. Broadly, the questionnaire comprised sections on general information, responsibility, communication, participation, satisfaction, and recommendations. A coupled group would be considered valid if the evaluation was successfully completed by both the resident student and the intern.

### Assessments

To assess the performance of resident students, both a pre-rotation examination and a rotation departmental examination adhering to the principles of the residency training program was conducted. Each examination comprised 50 multiple-choice questions assembled using the Teaching Assistant of People's Medical Publishing House app. These questions were designed to reflect real-life clinical scenarios, evaluating the comprehensive level of knowledge mastery of the resident students, including disease-related knowledge, diagnosis and differential diagnosis, analysis of ancillary examinations, clinical skills, communication and ethics.

To mitigate potential result bias, resident students in the same rotation period faced same questions in the pre-rotation examination and rotation departmental examination. However, due to variations in the rotation periods assigned to different resident students, the questions slightly varied between resident students for each iteration, and careful calibration was employed to ensure uniform difficulty levels across the different sets of questions. The examination was graded on a scale of 100 points, and the results were subsequently exported and subjected to analysis.

### Withdrawn

Throughout the duration of the coupled group, both resident students and interns had the freedom to withdraw from the arrangement if necessary. No withdrawn was declared during our experiment.

### Analysis

For categorical variable in Table 1, chi-square analysis was conducted by SPSS ver. 25.0. For continuous variables, student’s T test was performed and illustrated by GraphPad Prism 8. The Kolmogorov–Smirnov test was used to determine the examination score was distributed normally (*p* = 0.2253). The results are presented as Means ± Standard Deviations and as frequency (percentage) for categorical variables. Overall, *P*-value < 0.05 was considered statistically significant.

## Results

**Fig. 2:**
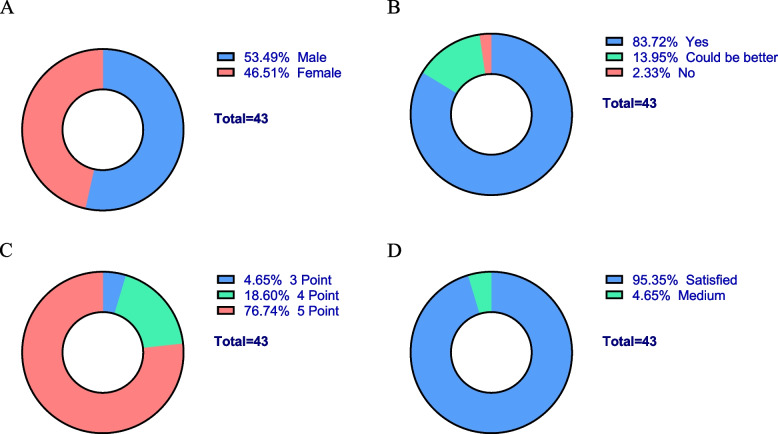
Feedback of interns in “one-to-one” policy as peer-students. The data was collected by questionnaire. **A** gender distribution. **B** assessment on whether resident students’ compliance with teaching obligations. **C** evaluation on sense of participation and belonging. The question was marked as 5 levels, 5- very good, 4- good, 3- medium, 2- poor, 1- very poor. **D** satisfaction of interns

*Satisfaction of undergraduates.* In total, all 43 interns enthusiastically participated in the "one-to-one" policy, demonstrating a strong interest in this novel approach to their clinical education. Among the participating interns, 23 (53.49%) were male, while 20 (46.51%) were female (Fig. 2A). Upon completion of their rotations, interns were requested to provide feedback by filling out a questionnaire, as detailed in the Methods section. The feedback received from the interns was overwhelmingly positive and highlighted the positive impact of the "one-to-one" policy. Remarkably, 36 interns (83.72%) acknowledged the sense of responsibility demonstrated by the resident students in their instruction. A total of 6 interns (13.95%) provided moderate evaluations along with valuable suggestions to further enhance the learning experience. Encouragingly, only one intern expressed dissatisfaction with the instruction provided by their coupled resident (Fig. 2B). Notably, the "one-to-one" policy was well-received by the interns, with 41 of them (95.35%) expressing high levels of satisfaction with the sense of engagement and belongingness fostered by this collaborative learning environment (Fig. 2C). Overall, the satisfaction rate of interns with the "one-to-one" policy reached an impressive 95.35% (Fig. 2D).

*Promotion of knowledge mastery on peer-teachers.* In the analysis of the rotation departmental examination, a total of 40 resident students were enrolled in the test group, while 118 resident students constituted the control group, as described previously.

**Fig. 3:**
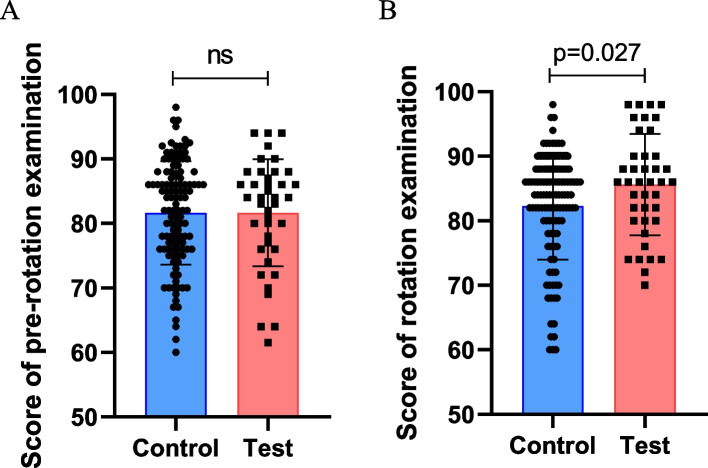
Effect of “one-to-one” policy on resident students. Scores of departmental examinations were represented by column and scatter image. **A** Pre-rotation examination. For control group (*n* = 117), 1 resident did not have valid score because of absence. **B** Rotation departmental examination. Error bar, SD. ns, no significance

Before the commencement of their rotations in our department, a pre-rotation examination was administered to resident students. The average score for the test group was 81.66 ± 8.325, while the control group obtained an average score of 81.66 ± 8.002 (Fig. 3A). There were no differences in pre-rotation examination scores between each group.

In the analysis of rotation departmental examination, the score of test group was 85.60 ± 7.886, while score of control group was 82.25 ± 8.292, *p* = 0.027 (Fig. 3B).

## Discussion

The transition from medical students to competent physicians is a critical phase in medical education. To further advanced their learning in clinic, we introduced the "one-to-one" policy in our study, aimed at fostering a collaborative learning environment between resident students and medical interns. In our study, the majority of peer-teachers were deemed responsible and satisfied based on intern feedback. Through a comparison of scores in both pre-rotation and departmental examinations, our finding suggests that participating in the "one-to-one" policy had a positive impact on the academic performance of voluntary resident students.

In a broader context, peer refers to individuals within the same academic year, however, resident students and interns are at different stage in their medical learning. Recently, near-peer signifies someone one or more years senior to another [15]. and near-peer assisted learning (NPAL) has also proven to be efficient in medical learning, benefiting both peer trainees and peer teachers [16]. Despite variations in grade levels between PAL and NPAL participants, the fundamental principle of mutual learning promotion remains consistent. Similarly, in Alkhail's research [15]. preclinical medical students took on the role of peer-students, while interns assumed the position of peer-teachers. As undergraduates progress to their residency after graduation, resident students coupled with interns were inspired by the interns, leading to improved academic performance compared to resident students in the control group. Therefore, our resident students-interns "one-to-one" policy is rooted in PAL or NPAL principles.

In modern medical education, competency-based learning is strongly emphasized [17]. Competency places a significant focus on outcomes, encompassing the ability to integrate knowledge, skills, values, and attitudes of physicians [18, 19]. While examination scores can reflect medical knowledge mastery, it is essential to recognize that they do not fully encapsulate the overall competency of resident students. Additional assessments, such as peer-teaching, collaborative abilities, and interpersonal communication skills [20]. should be conducted to comprehensively evaluate their development. In our design, the policy affords an opportunity for students with lesser medical knowledge to engage actively. As per the feedback gathered through questionnaires, the interaction of lower-grade students with the environment, along with their attempts to answer questions, enables peer-teachers to self-assess their understanding of various concepts. This process of self-inspection and self-education is cultivated. Moreover, in their dual roles as both a "senior physician" managing patients and an educator guiding students simultaneously, peer-teachers refine essential skills. This includes effective time management, enhanced communication skills, and heightened teaching awareness. We aspire that advancements in these aspects will not only enhance their immediate roles but also evolve into enduring assets, shaping them into exemplary physicians and medical educators throughout their careers.

Our study has limitations. First, our study centered on demonstrating the impact of the "one-to-one" policy on the performance of resident students as peer-teachers. While interns participated in the policy as peer-students, it would have been ideal to evaluate its effect on interns as well. Consequently, to comprehensively assess the impact of the policy on interns, a subsequent study with an expanded sample size is warranted. Additionally, there is a need for further measures to evaluate competency beyond examination scores. The voluntary nature of participation, constituting the inclusion criteria, implies that only active resident students engaged in our policy, potentially introducing selection bias. Furthermore, the presence of other confounding factors remains unclear due to the limited participant pool. While this study successfully demonstrates eligibility and the positive effects of the policy, future research employing a randomized design with a larger sample size could mitigate these biases. Lastly, it is essential to acknowledge and explore potential drawbacks of PAL or NPAL in subsequent studies. Despite minimal complaints from resident students about additional time and effort in participating in the policy, concerns raised by Xu et al. [21]. regarding a higher risk of burnout among resident students in PAL groups necessitate further investigation and consideration in future evaluations.

In conclusion, our study indicated that the "one-to-one" policy had a positive impact on interested resident students, empowering them as peer-teachers to interns and potentially promoting their competency in medical learning. The benefits of PAL and NPAL extend beyond peer-teachers to also include peer-students, fostering a dynamic learning environment that enhances the overall educational experience. As medical education continually evolves, embracing innovative teaching methodologies, can optimize the development of future physicians, equipping them to deliver exceptional patient care and meet the challenges of modern healthcare.

## Data Availability

The data that support the findings of this study are available from the corresponding authors, Prof. Wen Li or Prof. Jing Xue, upon reasonable request.
